# Occurrence of *Candida albicans* in Periodontitis

**DOI:** 10.1155/2021/5589664

**Published:** 2021-05-28

**Authors:** Brahim Jabri, Maryem Iken, Mohamed Achmit, Sana Rida, Oum Keltoum Ennibi

**Affiliations:** ^1^Research Laboratory in Oral Biology and Biotechnology, Faculty of Dental Medicine of Rabat, Mohammed V University in Rabat, Rabat, Morocco; ^2^Clinical Biology Department, Faculty of Medicine and Pharmacy, Mohammed V University in Rabat, Rabat, Morocco; ^3^Laboratory of Virology, Microbiology and Quality/ Eco-toxicology and Biodiversity, Faculty of Sciences and Techniques Mohammedia, University Hassan II Casablanca, Casablanca, Morocco; ^4^Department of Conservative Dentistry, Faculty of Dental Medicine of Rabat, Mohammed V University in Rabat, Rabat, Morocco; ^5^Department of Periodontology, Faculty of Dental Medicine of Rabat, Mohammed V University in Rabat, Rabat, Morocco

## Abstract

**Background:**

Periodontal diseases are the result of an imbalance between the microbiota and immune defense. The role of yeast in the pathogenesis of these diseases has been studied. This study aims to assess the occurrence of *Candida albicans* in periodontitis.

**Materials and Methods:**

Fifty subjects were recruited for the study (15 healthy individuals and 35 periodontitis subjects). The periodontal examination and plaque sampling were carried out for all patients. *Candida albicans* identification was based on culture, direct examination, and polymerase chain reaction. The statistical analysis was performed by SPSS 20 (SPSS Inc., Chicago, IL, USA).

**Results:**

Twenty percent of the diseased group harbored *Candida albicans* which was slightly higher than in the healthy group (7%), suggesting that, under normal conditions, yeast does not grow easily in subgingival sites. However, no significant difference between the healthy and periodontitis groups (*p*=0.23) was found. Our results also indicated that the presence of *Candida albicans* was neither gender nor age related in the studied groups.

**Conclusion:**

The results of this study suggest that *Candida albicans* occurs in periodontitis. More studies are needed to clarify the potential role of this yeast in different stages and forms of the disease.

## 1. Introduction

Among *Candida* species, *Candida albicans* is the most widespread yeast associated with healthy and pathologic oral conditions [1, 2]. Indeed, this opportunist microorganism belongs to commensal microflora in the healthy human digestive tract, but can become pathogenic under the influence of general or local favorable factors.

Periodontitis is a worldwide oral disease with a very complex etiopathogenesis, including dysbiosis and host immune responses that comaintain conditions for the occurrence of periodontal disease in susceptible individuals [3, 4]. Clinically, periodontitis involves attachment loss around teeth, forming periodontal pockets, and bone destruction [5, 6]. Several studies have raised the heterogeneity of the microflora associated with periodontal disease. Indeed, various microorganism species, including Gram-negative anaerobic bacteria organized in a complex biofilm, have been associated with the initiation and progression of periodontitis [7]. *Porphyromonas gingivalis*, *Tannerella forsythia*, and *Treponema denticola*, known as the “red complex” together with *Aggregatibacter actinomycetemcomitans* have been identified as the most periopathogenic bacteria associated with different forms of periodontitis worldwide [7, 8]. Lately, some studies discussed also the possible role of viruses [9, 10] and yeasts in periodontitis pathogenesis [11, 12]. Thus, as the disease breaks through, the periodontal pockets become the receptacle of a large number of microorganisms including *C. albicans* [6, 13, 14]. Nevertheless, the role of this yeast in the pathogenesis of periodontal disease remains unclear. The aim of the present study is to assess the presence of *Candida albicans* in periodontitis.

## 2. Materials and Methods

### 2.1. Study Population

Fifty subjects were recruited from the clinical department of Periodontology, “Center of Consultation and Dental Treatment,” Ibn Sina University Hospital of Rabat, Morocco (CCTD-CHIS, Rabat).

The approval for the study was obtained from the Research Ethics Committee of Mohammed V University, Rabat, Morocco (Ethical agreement number 102/19). All subjects were informed and signed the informed consent.

The inclusion criteria included patients in good general health, aged 18 years and above, with at least 20 teeth present, and who have not received any periodontal treatment or antibiotic medication during at least the previous six months prior to the study. The exclusion criteria included patients under orthodontic treatment, those who smoke or chew any other kind of tobacco, diabetic patients, those having symptomatic oral candidosis and/or had been under antifungal treatment, and pregnant and lactating women.

### 2.2. Methods

#### 2.2.1. Periodontal Examination

Prior to the periodontal examination, all patients signed the informed consent forms. The assessed clinical variables were modified gingival index (GI), plaque index (PI) [15], probing pocket depth (PPD), and clinical attachment loss (CAL). Probing pocket depth and attachment loss were measured using a standard periodontal probe (Hu-friedy, Chicago, IL), at six sites per tooth, i.e., distobuccal, buccal, mesiobuccal, distolingual, lingual, and mesiolingual, in all teeth excluding third molars. A set of full-mouth standardized intraoral radiographs was obtained from each patient.

Based upon clinical parameters, radiograph information, and patient age, we came to the clinical diagnosis based on the classification of the American Academy of Periodontology (AAP) [16].

Thus, in the healthy periodontium, there was no attachment loss, no periodontal pocket, no bone loss, and periodontal probing was ≤ 3 mm, whereas periodontitis was defined as having interdental clinical attachment lost detectable on at least 2 nonadjacent teeth or the presence of buccal or oral CAL ≥3 mm with pocketing ≥3 mm detectable at least in 2 teeth, but not associated with non-periodontitis-related causes (i.e., deep dental caries, CAL on a distal site of a second molar and associated with malposition or resulting from a third molar extraction, endodontic lesion draining through the marginal periodontium, and a vertical root fracture). Furthermore, periodontitis patients were classified by stage and grade based on the classification. Stages were defined based on severity (primarily periodontal breakdown and periodontitis-associated tooth loss) and complexity of management (pocket depth, infrabony defects, tooth mobility, furcation status, and masticatory disturbance). Grades of periodontitis were estimated based on direct or indirect evidence of progression rate in three categories: slow (grade A), moderate (grade B), and rapid progression (grade C).

#### 2.2.2. Plaque Sampling

A pool of subgingival biofilm samples were collected from four sites (one site per quadrant) in each patient. We selected the deepest site in each quadrant with a pocket depth equal or greater than 5 mm (PPD≥ 5 mm), clinical attachment loss ≥2 mm, and which were bleeding on probing.

Plaque sampling was obtained by introducing a sterilized absorbent paper point (30 mm diameter) into the gingival sulcus for 20 seconds (SURE DENT CORPORATION; CEO 197). The samples were placed in 2 mL of sterile phosphate buffered saline (PBS) as a transport medium and immediately transferred to the laboratory for analysis.

#### 2.2.3. Yeast Culture

Samples were first processed and cultured. They were dispersed and plated onto a sabouraud chloramphenicol medium for the isolation of yeasts. Plates were incubated at 37°C in non-CO_2_ atmosphere for 24 to 48 hours [17, 18]. The plates were checked daily for yeast growth.

**Figure 1:**
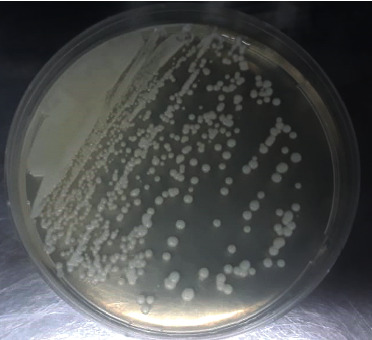
Macroscopic appearance of the *Candida* species on the sabouraud chloramphenicol culture medium.

Identification was based on colony and cellular morphology. *Candida albicans* colonies on the Sabouraud chloramphenicol medium are creamy whitish and smooth [19] (Figure 1).

**Figure 2:**
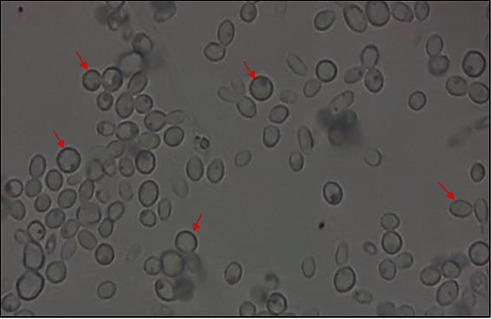
Appearance of yeast in a fresh culture (×40).

Under direct examination: *Candida* yeasts appear as oval, ovoid, or elongated and possibly budding elements [20] (Figure 2).

Pure cultures of yeast colonies were identified and confirmed later as *Candida albicans* by PCR.

#### 2.2.4. Identification of *Candida albicans* by PCR

Genomic DNA was extracted as follows: 4 to 6 yeast colonies were suspended in 1 ml of sterile 0.9% NaCl. The extraction was performed using a commercially available BIOLINE DNA extraction kit (ISOLATE II GENOMIC DNA KIT, USA) according to the manufacturer's instructions. The microtubes were centrifuged at 5000 g for 10 min. Then, the supernatant was removed and washed once with 2 mM EDTA (pH 8) and centrifuged for 10 min at 5000 g. The supernatant was discarded while the resulting pellet was stored. The extracted DNA was stored at −20°C for later use.

Molecular identification of the identified and purified yeast strains was accomplished by using the following specific primers for *C. albicans* detection, generating an amplicon of 500 base pairs (forward: 5′-TGCTTCAGTGTCAGTTATACCT-3′, 

Reverse: 5′-ACTGCTCAAACCATCTCTGG-3′) [20].

PCR amplification was performed in a 25 *μ*L reaction mixture containing the abovementioned primers (10 *μ*M each), 100 ng of extracted DNA, 100 *μ*M of each of the four deoxyribonucleoside triphosphates (dATP, dCTP, dGTP, and dTTP), 0.5 U of taq DNA polymerase, and 5 mM of buffer.

The PCR amplification parameters were as follows: an initial denaturation of 1 min at 95°C, followed by 35 cycles of denaturation of 15 seconds at 95°C, a hybridization of 20 seconds at 50°C, and an elongation of 15 seconds at 72°C, with a final elongation of 3 min at 72°C. The PCR products were analyzed by 1% agarose gel electrophoresis in the presence of a Dna Ladder 200 bp (Bioline) and visualized by using a UV transilluminator system G-Box (Syngene™).

#### 2.2.5. Statistical Analysis

The statistical analysis was performed with a statistical program SPSS 20 (SPSS Inc., Chicago, IL, USA). For each group, continuous variables with normal distribution were presented as mean ± standard deviation (age, depths of periodontal pockets, and attachment loss), and categorical variables were given as percentage. Chi-square Pearson's test was performed to compare the variables between two independent groups, and Student's *t* test was performed to compare the means of the independent samples. The statistical significance threshold used was *p* < 0.05.

## 3. Results

Fifty subjects accepted to participate to the study and fully fit the inclusion criteria. Thirty-seven (74%) among this population were female, and thirteen (26%) were male. The average age was 31 ± 11 years. Fifteen individuals aged between 21 and 52 years were healthy, and 35 patients aged between 18 and 62 years were identified as having periodontitis; however, there was no statistically significant difference between the two groups. Demographic and clinical characteristics of the study population are summarized in Table 1.

In the periodontitis group, subgroups were identified regarding staging and grade. Thus, the patients were diagnosed as having periodontitis stage II or III and grade B or C. The statistical analysis showed no significant difference between periodontitis stage II and III regarding the average age. However, age was lower in patients who had grade C periodontitis in comparison with those who had grade B, and the difference was statistically significant (Tables 2 and 3).

**Figure 3:**
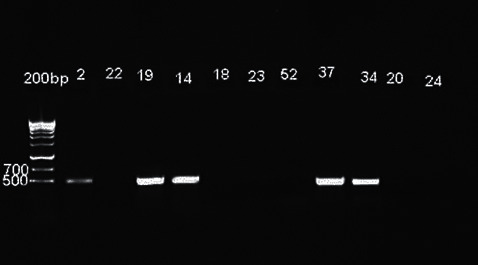
Agarose gel electrophoresis of PCR products of *Candida albicans* isolates.

The growth of yeast colonies was recorded as a positive growth and the subject as a positive carrier. Among the 17 positive cultures, height isolates were identified as *C. albicans* by PCR (one healthy subject and 7 periodontitis patients) (Figure 3).

The presence of *C. albicans* was studied among the periodontitis population according to periodontal status. No significant differences were found between different groups (Table 4).

Analysis of the presence of *Candida albicans* between subgroups in the periodontitis group did not show any statistical difference neither when comparing stages II and III (33.3% and 17.2%, respectively, *p*=0.370) nor when comparing grades Band C (21.0% and 18.7%, respectively, *p*=0.865).

## 4. Discussion

Microbial-flora-associated periodontitis is very complex and has been continuously studied for many years. However, it should be emphasized that even if bacteria are considered as a primary agent in periodontal pathogenesis, other microorganisms, e.g., viruses and yeasts, are increasingly involved in the microbial etiology of periodontal diseases.

*Candida albicans*, the most widespread yeast in the oral cavity, can be isolated in healthy subjects without any clinical manifestation. It colonizes commonly the tongue [1]. However, many studies reported its presence in the healthy periodontium too. In the present study, 7% of the periodontal healthy patients harbored *C. albicans* in the subgingival plaque. These results are high in comparison to those reported by Urzúa et al. [21], which reported a prevalence of 3.57% in a population of 28 healthy subjects [21]. Meanwhile, Canabaro et al. [22] reported that 14.28% of 20 examined healthy subjects were yeast positive. Other studies showed variable occurrence (16% to 36%) of *C. albicans* in the subgingival plaque of the healthy periodontium [11, 23–25]. In immunocompetent subjects, *C. albicans* exists as a minor component of the oral biofilm [26]. It has been suggested also that its presence in the subgingival area could be transient [27]. However, it can also exist in periodontal pockets, and its role in periodontal pathogenesis is not yet clear.

In the present study, 20% of periodontitis patients were positive for *C. albicans*. Almost the same result had been found by Dahlén [28], who reported a prevalence of 17% and suggested that this yeast was more common in periodontitis patients than in healthy people. Nevertheless, no significant differences were found between these two groups (*p* > 0.05) in the present study.

Relatively few studies have analyzed the possible role of *Candida albicans* in periodontitis [23, 24, 27]. Matić Petrović et al. [25] reported that there is no impact of periodontal pocket depth on the presence of subgingival yeast. However, it should be pointed out that the mean probing depth in their study was 2.89 ± 0.944 for periodontitis patients compared to 2.02 ± 0.524 as the mean probing depth in healthy periodontium subjects.

Many studies showed an association between subgingival colonization by yeast subspecies, including *Candida albicans*, and the presence of severe periodontitis [6, 28]. Jarvensivu et al. [29] showed a prevalence of 16% in a population of 25 patients with chronic periodontitis. *C. albicans* remains the most common fungal pathogen found in patients with periodontitis [21, 22]. In the present study and according to the new classification of periodontal diseases and conditions, the periodontitis group included 2 distinguished subgroups based onstage case definition: stage II and stage III. No statistical difference was seen between stage II and stage III, regarding neither age nor the presence of *Candida albicans*. When comparing the periodontitis group based on grade, two subgroups were distinguished, a grade B group and grade C group. A significant difference was found for the average age between the two groups. Indeed, patients with grade C periodontitis were younger, and the estimate rate of periodontitis progression was rapid in comparison with group B periodontitis patients. However, there was no significant difference between these grade subgroups when considering the presence of *Candida albicans*. Thus, the present study suggests that the presence of *Candida albicans* in the periodontitis group seems not to be related to the stage or the grade. Nonetheless, these results must be considered with caution because of the small size of the study population. Moreover, all included patients were subjects seeking treatment in a hospital structure. Thus, further well-designed study, including a bigger size population, is needed to assess and understand the real contribution of *Candida albicans* in the modified microbiome associated with periodontitis.

The role of *C. albicans* in periodontitis pathogenesis is yet unclear. Indeed, this yeast could alter the oral microbiome and, therefore, influence significantly bacteria colonization [30, 31]. Coadherence between *Candida albicans* and some bacteria may help the formation of complex biofilms with mixed species affecting, therefore, the microbial pathogenesis; in [26, 32, 33], it was suggested also that *C. albicans* promotes bacterial invasion of host cells by anaerobic bacteria such as *P. gingivalis* and, thus, induces infections by anaerobic bacterial diseases.

In addition, *C. albicans* can grow either aerobically or anaerobically [34], which may explain their presence in deep periodontal pockets [22, 35]. Other studies reported that *C. albicans* is capable of adhering to epithelial cells and induce inflammation [36]. *C. albicans* may have some virulence factors such as aspartyl proteinase (SAPs), phospholipases, and exoenzymes which disturb the locally immune response by the inhibition of polymorphonuclear neutrophil phagotocytosis and induce indirectly inflammatory reactions [24, 36, 37].

When comparing the presence of *C. albicans* regarding gender, 19% and 8%, respectively, of women and men were yeast positive. These results are the same as those reported by Reynaud et al. [24] who showed a prevalence of 20.3% among women compared to 8.2% in men. Nevertheless, as shown previously by Canabaro et al. [22], the presence of *Candida albicans* is not gender related (*p*=0.342).

## 5. Conclusions

*Candida albicans* was present in periodontitis patients in this study. However, no statistical differences were found between the studied groups. Even if the role of *C. albicans* in periodontal disease has not yet been established, this yeast is considered an important pathogen in the progression and persistence of this disease. Thus, it would be interesting to study in depth the virulence factors and carry out in vitro studies to better understand the potential role of this yeast in this pathology and, especially, in refractory periodontal diseases.

## Figures and Tables

**Table 1:** Demographic and clinical characteristics of the study population.

	Healthy (*n* = 15; 30%)	Periodontitis (*n* = 35; 70%)	Total (*n* = 50; 100%)	*p* value
Gender (male/female)	5/10	8/27	13/37	*p*=0.439
Age (years) (mean ± SD)	28.73 ± 9.24	33.31 ± 12.16	31.94 ± 11.47	*p*=0.199
Plaque index (mean ± SD)	0.21 ± 0.10	2.41 ± 0.69	1.75 ± 1.17	*p* ≤ 0.001
Gingival index (mean ± SD)	0.05 ± 0.04	2.48 ± 0.68	1.75 ± 1.26	*p* ≤ 0.001
Pocket depth (mean ± SD)	2.80 ± 0.35	5.79 ± 1.83	4.90 ± 2.07	*p* ≤ 0.001
Clinical attachment loss (mean ± SD)	—	4.61 ± 1.24	—	—

**Table 2:** Comparison of clinical parameters in the periodontitis group according to stages II and stage III.

	Periodontitis group (*n* = 35)		
	Stage II (*n* = 6; 17%)	Stage III (*n* = 29; 83%)	*p* value
Age (mean ± SD)	33.00 ± 7.23	33.38 ± 13.04	0.946
Periodontal pockets depths (PPD) (mean ± SD)	5.00 ± 0.44	5.96 ± 1.97	0.251
Clinical attachment loss (CAL) (mean ± SD)	3.17 ± 0.2	4.91 ± 1.15	0.001

**Table 3:** Comparison of clinical parameters in the periodontitis group according to grade B and grade C.

	Periodontitis group (*n* = 35)		
	Grade B (*n* = 19; 54%)	Grade C (*n* = 16; 46%)	*p* value
Age (years) (mean ± SD)	41.89 ± 8.73	23.13 ± 6.31	0.000
Periodontal pockets depths (PPD) (mm) (mean ± SD)	4.78 ± 1.39	7.00 ± 1.56	0.000
Clinical attachment loss (CAL) (mm) (mean ± SD)	4.12 ± 0.73	5.20 ± 1.47	0.008

**Table 4:** The presence of *Candida albicans* among the study population according to periodontal status.

		*Candida albicans*		*p* value
		Negative carriers (*n* = 28; 80%)	Positive carriers (*n* = 7; 20%)	
Gender	Female	21 (78%)	06 (22%)	0.484
	Male	07 (87.5%)	01 (12.5%)	
Age (years) (mean ± SD)		32.02 12.19	31.50 7.07	0.907
Plaque index (mm) (mean ± SD)		2.37 ± 0.75	2.59 ± 0.34	0.460
Gingival index (mm) (mean ± SD)		2.47 ± 0.74	2.51 ± 0.39	0.907
Periodontal pocket depth (PPD) (mm) (mean ± SD)		6.07 ± 1.87	4.68 ± 1.20	0.072
Clinical attachment loss (mm) (mean ± SD)		4.81 ± 1.29	3.82 ± 0.55	0.059

## Data Availability

All data used to support the findings of the study are included in the article.
